# Gastropleurocutaneous fistula secondary to cement prosthesis migration: A rare late complication of chest wall sarcoma reconstruction

**DOI:** 10.1186/s12893-025-03312-x

**Published:** 2025-11-17

**Authors:** Mohammad Alaa Aldakak, Nawwar Fallouh, Bassel Ibrahim, Ahmad Al Dalati, Yousef Al Jabban, Bassam Darwish, Yehia Taifour

**Affiliations:** 1 Faculty of Medicine, Damascus University, Damascus, Syrian Arab Republic; 2 Faculty of Medicine, Al-Mouwasat University Hospital, Damascus University, Damascus, Syrian Arab Republic

**Keywords:** Gastropleurocutaneous fistula, Chest wall reconstruction, Cement prosthesis migration, Thoracoabdominal fistula, Polymethylmethacrylate (PMMA), Diaphragmatic repair, Chest wall sarcoma, Surgical complication, Prosthesis erosion, Case report

## Abstract

**Background:**

Gastropleurocutaneous fistula (GPCF) is a rare, potentially life-threatening condition defined by an abnormal connection between the stomach and the pleural cavity with an accompanying cutaneous tract. It is distinct from a gastropleural fistula, which lacks an external cutaneous extension. To our knowledge, no prior reports have described GPCF caused by migration and erosion of a cement chest-wall prosthesis.

**Case presentation:**

We present the case of a 40-year-old female with a history of anterior chest wall sarcoma resection and cement prosthesis reconstruction performed 23 years earlier. The patient developed a chronic cutaneous discharge beneath the left breast and was ultimately diagnosed with a gastropleurocutaneous fistula. Although she had experienced prior episodes of discharge, this was the first time the condition was confirmed through imaging and intraoperative findings. Imaging revealed a large left thoracoabdominal collection and erosion of the diaphragm by the prosthesis into the gastric fundus. Surgical management included isolation and stapling of the gastric fistula, diaphragmatic repair using polypropylene mesh, and pleural cavity debridement.

**Case discussion:**

The development of gastropleurocutaneous fistula secondary to cement prosthesis migration has not been previously documented. While cement-based prostheses offer structural stability, long-term complications such as erosion into visceral organs may occur, particularly with inadequate fixation or soft tissue coverage. Diagnosis requires a high index of suspicion and a combination of radiological and endoscopic modalities. Surgical repair remains the definitive treatment, often requiring a combined thoracoabdominal approach.

**Conclusion:**

This case highlights a rare but serious late complication of chest wall reconstruction with cement prosthesis. It underscores the need for long-term surveillance in patients with synthetic implants and the importance of individualized surgical planning in managing complex thoracoabdominal fistulas.

## Introduction

Gastropleurocutaneous fistula (GPCF) is an exceptionally rare entity defined by an abnormal communication between the stomach and the pleural cavity with a cutaneous tract opening to the skin [1]. The exact mechanism behind the formation of GPCF remains unclear; however, it is likely a consequence of a postoperative leak that leads to the development of an abscess [2]. The clinical presentation of GPCF is typically insidious, with patients often presenting with chronic, non-specific respiratory symptoms such as cough and shortness of breath [2]. In addition to malignancy, GPCF can also result from various other causes. It has been shown to be associated with chest-abdominal trauma or complications following surgeries involving the lungs, diaphragm, or gastrointestinal organs, such as lung resection or diaphragmatic hernia repair [3]. Cement-based prostheses are commonly used in chest wall reconstruction for structural support but may cause complications such as loosening, infection, or erosion, especially with poor fixation or compromised tissue [4, 5]. GPCF is a diagnostically challenging condition, often requiring imaging and endoscopy for confirmation. The presence of gastric contents in pleural drainage is a key diagnostic clue [6, 7]. Management of GPCF is complex and often requires surgical intervention, as conservative approaches frequently fail due to high complication rates. Definitive treatment typically involves a combined thoracoabdominal approach for gastric resection, diaphragmatic repair, and pleural drainage [8, 9]. We report a case of a 40-year-old female presenting with a GPCF resulting from a previous surgical intervention.

## Case presentation

**Fig. 1:**
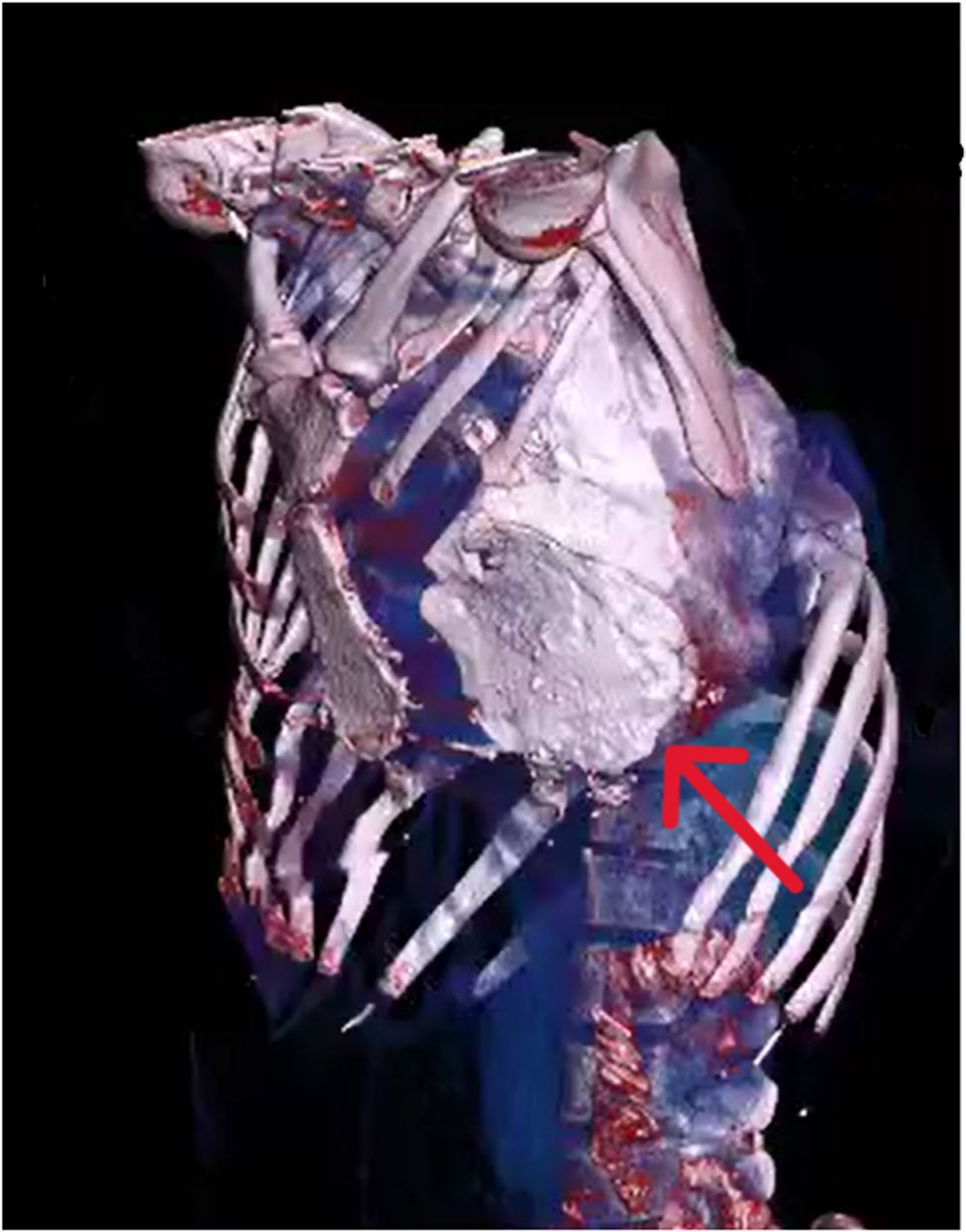
Cutaneous fistula opening located in the left lower thoracic region (red arrow), showing inflamed surrounding skin with erythema and epithelial breakdown. The opening is positioned along an old surgical scar

A 40-year-old female, non-smoker and non-alcoholic, presented to Al-Mouwasat University Hospital with a chronic history of purulent and food-like discharge from a cutaneous opening located beneath the left breast, directly over a previous surgical scar **[**Figure 1**]**.

Approximately 23 years prior to presentation, the patient underwent a major surgical resection of a left anterior chest wall sarcoma following seven cycles of chemotherapy. The surgery involved resection of ribs 4 to 6 along the left midclavicular to anterior axillary line, along with partial resection of the left lung, followed by reconstruction using a cement prosthesis. The patient did not receive adjuvant radiotherapy following her initial chest wall sarcoma resection. Additionally, no radiological or intraoperative evidence of radiation-induced tissue changes, such as fibrosis or necrosis, was observed during the current evaluation. Histopathological reports from the patient’s previous oncologic and surgical interventions were unavailable, as these procedures were performed at external institutions more than a decade ago. Around 12 years after the initial surgery, the patient began to experience intermittent purulent discharge from the posterior left upper back, just below the scapula. Surgical drainage and debridement were performed at an outside facility, but the discharge persisted in a relapsing pattern.

Approximately 4 months prior to admission, the patient developed abdominal pain associated with discharge of purulent and partially digested material from the old thoracic surgical scar. She underwent a laparotomy at an outside facility, during which a perforation in the stomach wall was identified, along with a retained foreign body (presumably part of the cement plate). The gastric defect was sutured, but the discharge continued postoperatively.

On presentation, her vital signs were (BP: 120/70 mmHg, HR: 120 bpm, Temp: 37 °C, SpO₂: 95%). Laboratory workup revealed significant leukocytosis (WBC: 32,000/mm³), elevated CRP (90 mg/L), hypoalbuminemia (2.6 g/dL), and anemia (Hb: 8.4 g/dL), consistent with ongoing chronic inflammation and malnutrition.

**Fig. 2:**
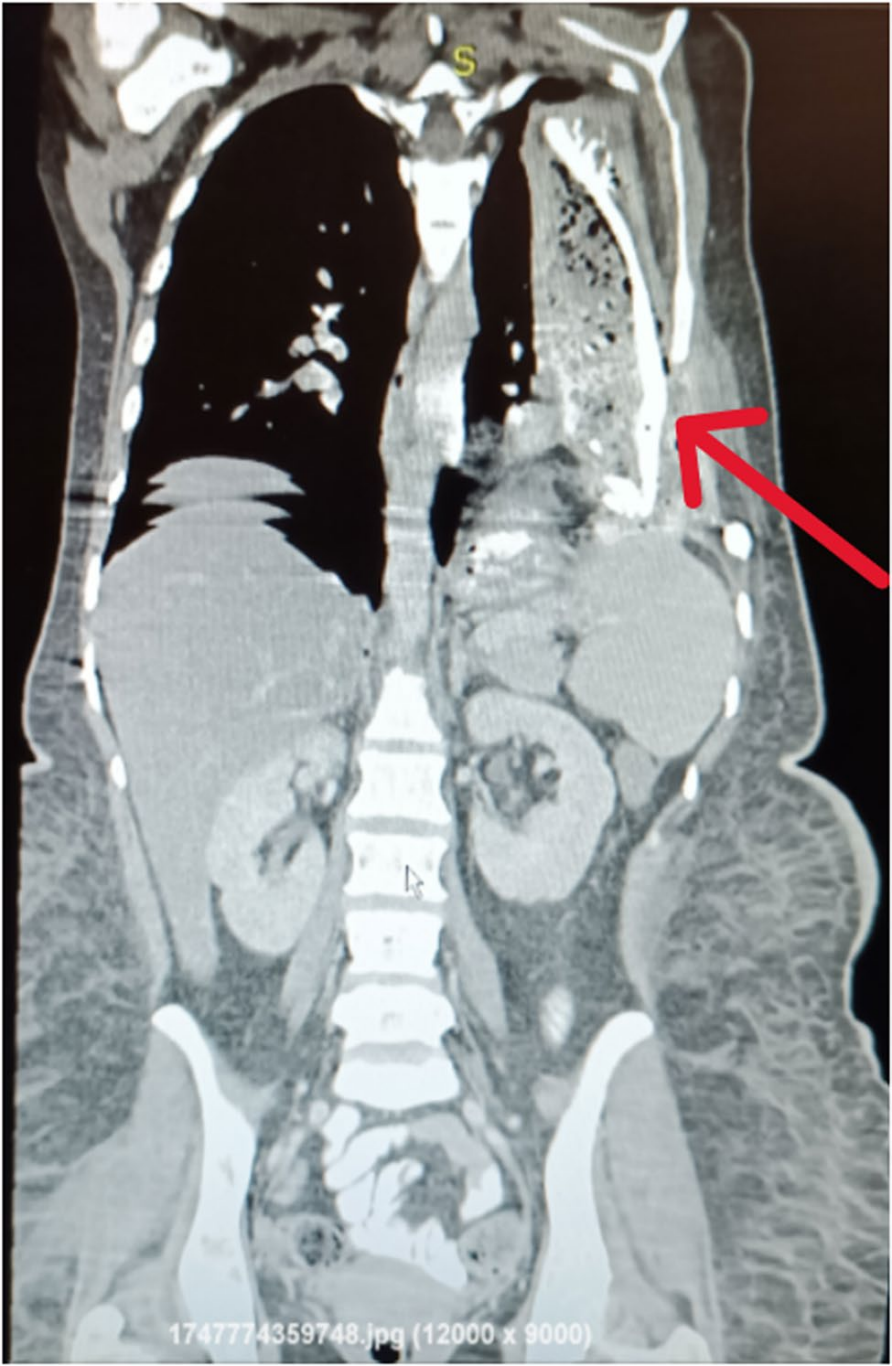
Coronal contrast-enhanced CT image showing a complex left-sided collection (red arrow) extending from the upper abdomen into the thoracic cavity. The collection contains air and fluid components and communicates with the anterior abdominal wall. Discontinuity of the diaphragm is also noted

**Fig. 3:**
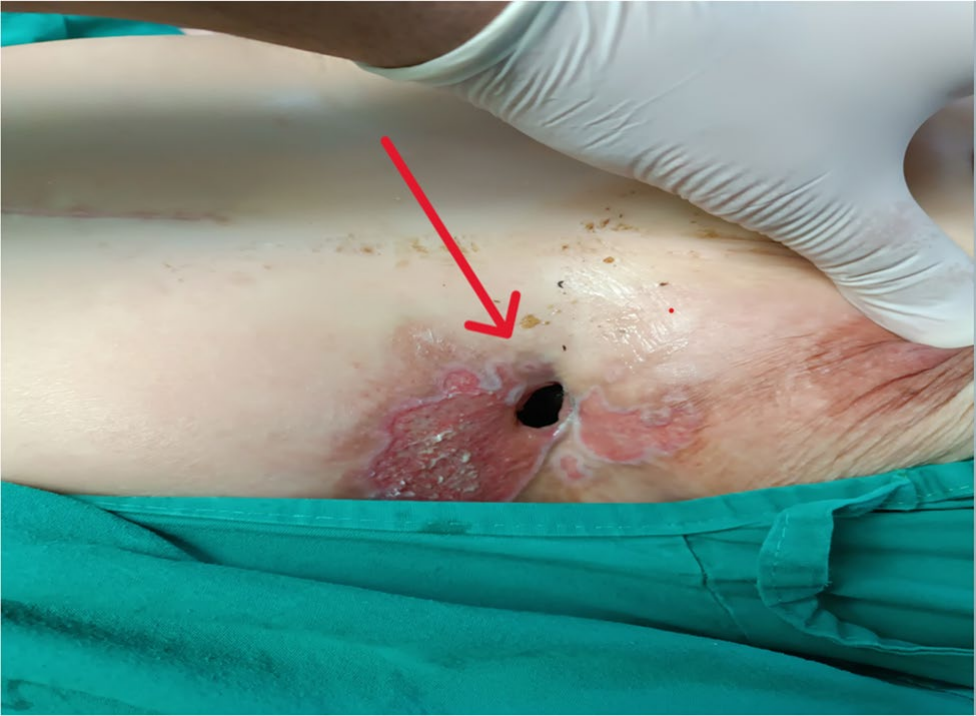
Oblique 3D CT reconstruction emphasizing the spatial relationship between the cement prosthesis (red arrow), missing ribs, and remaining thoracic cage. The prosthesis appears adherent to surrounding structures with evidence of chronic structural deformation

Chest radiography showed dense opacity in the left hemithorax with absence of ribs 4 through 6. Contrast-enhanced CT of the chest and upper abdomen demonstrated a large heterogeneous left-sided thoracoabdominal collection with gas and fluid, extending across a disrupted left hemidiaphragm into the thoracic cavity and communicating with the anterior thoracic wall via a cutaneous fistula—findings consistent with a chronic GPCF with secondary cutaneous extension. Post-surgical changes were evident, including absence of left ribs 4–6 and chest wall distortion **[**Figure 2**].** Three-dimensional CT reconstructions further delineated these abnormalities, revealing a large cement prosthesis embedded in the thoracic wall and providing valuable anatomical detail for surgical planning **[**Figure 3**]**.

**Fig. 4:**
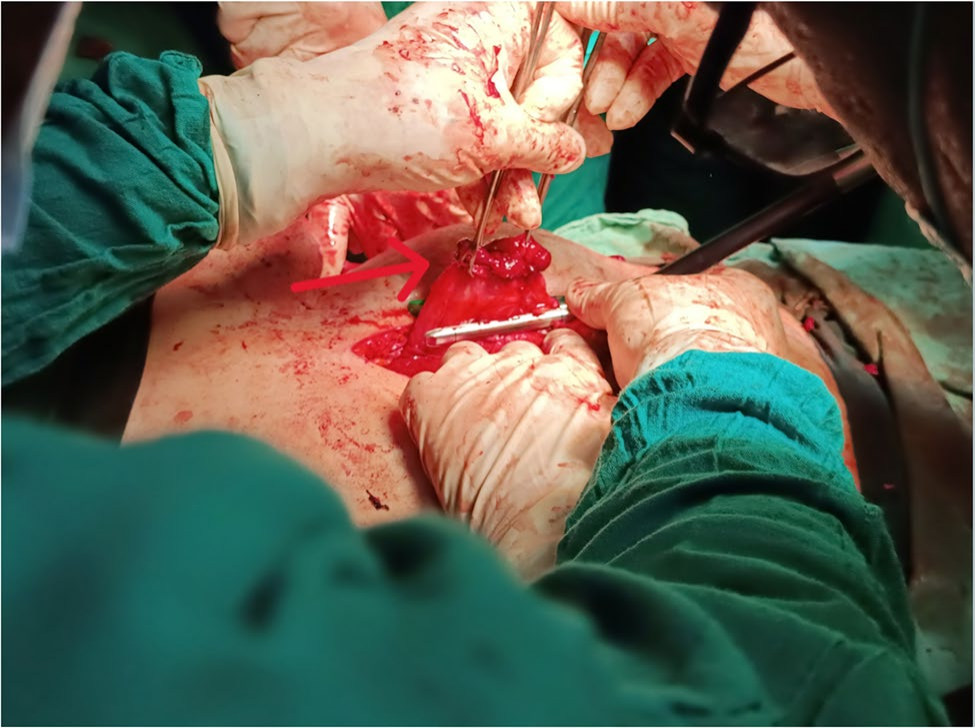
Intraoperative view showing dissection of the fistulous tract (red arrow) between the gastric fundus and thoracic wall. A linear stapler is applied for fistula closure following complete isolation

The patient underwent exploratory laparotomy. A midline upper and lower abdominal incision was performed. Dense intra-abdominal adhesions were encountered; the stomach was found to be firmly adherent to the diaphragm. Dissection exposed a fistulous tract extending from the gastric fundus to the left pleural cavity, confirmed using a metallic surgical probe. The gastric fistula was isolated and stapled **[**Figure 4**]**.

A large diaphragmatic defect was identified. A midline abdominal incision was made extending from the epigastrium to the lower abdomen. The diaphragmatic defect was repaired using an 11 × 7 cm macroporous polypropylene mesh. The mesh was fixed to the diaphragmatic rim using interrupted non-absorbable monofilament sutures, ensuring a tension-free closure.

The thoracic fistulous tract was debrided, the pleural cavity was irrigated, and multiple drains were placed in the pelvic, subhepatic, and perisplenic regions. The cement prosthesis was not removed during this operation due to its dense integration with the chest wall and risk of structural instability. Surgical efforts focused on isolating the fistula and repairing the diaphragmatic defect.

The patient remained hospitalized for 7 days postoperatively. Drain output progressively decreased and remained non-purulent. Oral intake was gradually resumed, and nutritional status improved with supportive care and dietary supplementation. At six-month follow-up, the patient remained clinically well with no recurrent drainage or infection; oral intake was normal, and interval imaging showed no recurrent collection or fistulous communication.

## Discussion

Gastropleurocutaneous fistula GPCF refers to an abnormal connection between the stomach and the pleural cavity with an accompanying cutaneous tract [10]. Gastropleurocutaneous fistula GPCF requires both a gastropleural communication and a cutaneous tract opening to the skin. In contrast, a gastropleural fistula (GPF) involves a communication between the stomach and the pleural space without an external cutaneous extension. Recognizing this distinction guides evaluation (including assessment of cutaneous drainage pathways) and operative planning, which often requires addressing thoracic, diaphragmatic, gastric, and cutaneous components. It is most frequently reported as a complication of peptic ulcer disease, thoracoabdominal trauma, subdiaphragmatic abscesses, empyema, malignancies, surgical interventions, or chemoradiotherapy [10]. The condition was first described by Markowitz and Herter in 1960 as a rare complication following rupture of a hiatal hernia [11]. Subsequent reports have linked GPCF to chest-abdominal trauma and postoperative complications involving thoracic or abdominal structures, such as after lung resections or diaphragmatic hernia repairs [11–13]. Cement prostheses, particularly those composed of polymethylmethacrylate (PMMA), have been utilized in orthopedic oncology for structural reconstruction following tumor resections. They can be molded intraoperatively to fit the defect and provide immediate anatomical support. While they help preserve chest wall contour and protect underlying structures, these prostheses do not integrate biologically with host bone and may lead to long-term complications such as pseudarthrosis, mechanical erosion, or infection. These risks are particularly relevant in long-surviving patients or when used in contaminated fields with limited soft tissue coverage [5]. The use of cement prostheses in chest wall reconstruction, especially following resection of chest wall sarcomas, has been associated with complications such as prosthesis loosening, soft tissue necrosis, infection, and even direct cardiac or visceral injury due to mechanical friction with adjacent structures. These events were largely attributed to inadequate fixation or insufficient soft tissue coverage [4]. We hypothesize that the fistula formation in our case was likely multifactorial, involving chronic low-grade inflammation initiated by the cement prosthesis acting as a foreign body, possibly exacerbated by intermittent low-level infections. Mechanical erosion of the diaphragm over time, combined with the absence of adequate soft tissue coverage, likely facilitated progressive penetration into the stomach wall. While it is difficult to establish definitive causality in a single case, the absence of evidence of tumor recurrence or radiation-induced injury, combined with the direct anatomical contact observed between the cement prosthesis and the gastric fundus, strongly suggests that prosthesis-induced mechanical erosion was the primary contributor to fistula formation. Although such complications are infrequent, long-term outcomes remain uncertain, as cement prostheses do not achieve true osseous fusion and may result in pseudarthrosis over time [5]. In contrast to the generally favorable outcomes reported in a clavicle reconstruction series—where all implants remained intact without infection or rejection [5]—our case represents a unique and severe late complication: a displaced cement prosthesis progressively eroding through the diaphragm into the stomach, ultimately forming a GPCF with cutaneous extension, while the prosthesis itself remained in place. Although the cement prosthesis appeared to be chronically contaminated, complete removal was not feasible due to dense adherence to the chest wall and lack of adequate reconstructive alternatives at the time. To minimize the risk of recurrence, we implemented extensive local debridement, ensured adequate drainage, and initiated a prolonged course of broad-spectrum antibiotics with outpatient follow-up. Regular clinical and radiological surveillance is planned. Diagnosing GPCF is often difficult due to its broad range of causes and variable clinical features [6]. Imaging modalities such as chest X-ray, computed tomography (CT), and upper gastrointestinal (GI) series are essential tools for establishing the diagnosis [6, 14, 15]. The most frequent clinical signs include upper abdominal discomfort, left-sided chest pain, hydropneumothorax, and, in some cases, tension pneumothorax [16]. Suspicion should be heightened when gastric secretions, food particles, or bile are observed through a thoracostomy tube [7]. Final diagnosis typically relies on a combination of contrast radiography, upper GI endoscopy, and analysis of pleural fluid contents [17]. Management of GPCF often begins conservatively, but surgical intervention is frequently required due to the high rate of complications, including mortality in up to 33% of cases. Surgical options include segmentectomy or lobectomy, and successful outcomes have been reported using both open and laparoscopic techniques. Definitive repair typically involves a combined thoracic and abdominal approach with partial gastric resection, diaphragmatic repair, and pleural washout with drainage. Endoscopic closure using vicryl mesh and fibrin glue has also been described [8, 9]. The decision to use a permanent synthetic polypropylene mesh, despite the contaminated field, was based on the large size of the diaphragmatic defect (11 × 7 cm) and the lack of suitable absorbable or biological alternatives. The prosthesis was positioned away from the gastric and pleural cavities after thorough irrigation, and the mesh was placed with wide overlap after thorough irrigation and debridement. While absorbable meshes may reduce infection risk, they offer limited long-term tensile strength, which was crucial in this case. The patient’s stable postoperative course and lack of recurrent infection support this choice. Nonetheless, determining the optimal timing for surgery remains challenging, and must be tailored to each patient’s clinical course [10].

## Conclusion

To our knowledge, no prior reports have described cases of a GPCF caused by migration and erosion of a cement prosthesis used in chest wall reconstruction. Given the increasing use of synthetic materials in oncologic surgery, clinicians should remain vigilant for delayed complications, and long-term follow-up should be considered an integral part of postoperative care.

## Learning points

- Cement prostheses, though structurally effective, may lead to serious long-term complications such as fistula formation.
- Persistent cutaneous discharge in patients with prior chest wall reconstruction warrants evaluation for deeper visceral communication.
- A multidisciplinary approach and long-term follow-up are critical in managing complex thoracoabdominal fistulas.

## Data Availability

All data generated or analyzed during this case report are included in this published article and its supplementary information files.

## Abbreviations

BP: blood pressure
CRP: C-reactive protein
CT: computed tomography
3D: three-dimensional
GI: gastrointestinal
GPF: gastropleural fistula
GPCF: gastropleurocutaneous fistula
Hb: hemoglobin
HR: heart rate
IRB: institutional review board
PMMA: polymethylmethacrylate
SpO₂: peripheral capillary oxygen saturation
WBC: white blood cell count

## References

[1] Al-Shurafa H, Alghamdi S, Albenmousa A, Alolayan H, Al-Shurafa Z. Gastropleurocutaneous fistula after single anastomosis gastric bypass: A case report and review of the literature. Int J Surg Case Rep. 2017;35:82–6. 10.1016/j.ijscr.2017.03.035. 28458144 10.1016/j.ijscr.2017.03.035 PMC5412257

[2] Nguyen D, Dip F, Hendricks L, Lo Menzo E, Szomstein S, Rosenthal R. The surgical management of complex fistulas after sleeve gastrectomy. Obes Surg. 2016;26(2):245–50. 10.1007/s11695-015-1788-2. 26224371 10.1007/s11695-015-1788-2

[3] Prasertsan P, Anuntaseree W, Ruangnapa K, Saelim K. Gastropleurocutaneous fistula masquerading as chylothorax in a child with lymphoma. BMJ Case Rep. 2019;12(7):e228987. 10.1136/bcr-2018-228987. 31296637 10.1136/bcr-2018-228987 PMC6626480

[4] Jönsson P, Gyllstedt E, Hambraeus G, Lillogil R, Rydholm A. Chest wall sarcoma: outcome in 22 patients after resection requiring thoracic cage reconstruction. Sarcoma. 1998;2(3–4):143–7. 10.1080/13577149877894. 18521246 10.1080/13577149877894 PMC2395395

[5] Lin B, He Y, Xu Y, Sha M. Outcome of bone defect reconstruction with clavicle bone cement prosthesis after tumor resection: A case series study. BMC Musculoskelet Disord. 2014;15:183. 10.1186/1471-2474-15-183. 24885109 10.1186/1471-2474-15-183 PMC4046063

[6] Luo RB, Liu S, DeLong JC, Jacobsen GR, Sandler BJ, Horgan S. Laparoscopic gastropleurocutaneous fistula repair: A minimally invasive solution for a complex problem. J Laparoendosc Adv Surg Tech A. 2017;27(4):416–9. 10.1089/lap.2016.0569. 28080207 10.1089/lap.2016.0569

[7] Armstrong RL, Heyse PB. Gastropleurocutaneous fistula in metastatic ovarian cancer. J Surg Case Rep. 2014;2014(5):rju033. 10.1093/jscr/rju033. 24876505 10.1093/jscr/rju033 PMC4024617

[8] Sersar SI, Maghrabi LA. Respiratory-digestive tract fistula: Two-center retrospective observational study. Asian Cardiovasc Thorac Ann. 2018;26(3):218–23. 10.1177/0218492318755013. 29392975 10.1177/0218492318755013

[9] Fong DG, Jajoo K, Piesman M, Lautz DB, Thompson CC. Endoscopic repair of gastropleural and gastroperitoneal fistulas with layered Vicryl and fibrin glue. Gastrointest Endosc. 2007;65(5):AB275.

[10] Shono Y, Sekioka A, Ito T, Tsuboi K, Ota S. A rare case of gastropleurocutaneous fistula during chemotherapy for metastatic colorectal cancer. Cureus. 2025;17(3):e79937. 10.7759/cureus.79937. 40177432 10.7759/cureus.79937 PMC11962330

[11] Markowitz AM, Herter FP. Gastro-pleural fistula as a complication of esophageal hiatal hernia. Ann Surg. 1960;152(1):129–34. 10.1097/00000658-196007000-00018. 14421290 10.1097/00000658-196007000-00018 PMC1613612

[12] Lakshminarayanan B, Morgan RD, Platt K, Lakhoo K. A leak too far–gastro-pleural fistula mimicking recurrence of repaired congenital diaphragmatic hernia following fundoplication. J Radiol Case Rep. 2013;7(9):33–8. 10.3941/jrcr.v7i9.1505. 24421956 10.3941/jrcr.v7i9.1505 PMC3888184

[13] Chowdary PB, Sadashivaiah SB, Gangappa RB, Shivashankar SC. Gastro pleural fistula: A rare entity presenting as a complication of empyema thoracis following stab injury to the chest. J Clin Diagn Res. 2015;9(4):PD05–6. 10.7860/JCDR/2015/12257.5764. 26023592 10.7860/JCDR/2015/12257.5764 PMC4437108

[14] Baka N, Batra V, Yeung V, Lin S. Diagnosis and management of gastro-pleural fistula in metastatic malignancy. Cureus. 2019;11(4):e4455. 10.7759/cureus.4455. 31205841 10.7759/cureus.4455 PMC6561524

[15] Iqbal SM, Zhi C, Masud M, Aslam HM, Qadir MA. Gastropleurocutaneous fistula: A rare complication of a common procedure. Cureus. 2019;11(2):e4136. 10.7759/cureus.4136. 31058019 10.7759/cureus.4136 PMC6485830

[16] Warburton CJ, Calverley PM. Gastropleurocutaneous fistula due to gastric lymphoma presenting as tension pneumothorax and empyema. Eur Respir J. 1997;10(7):1678–9. 10.1183/09031936.97.10071678. 9230265 10.1183/09031936.97.10071678

[17] Chow H, Jung A, Talbott J, Lin AM, Daud AI, Coakley FV. Tumor fistulization associated with targeted therapy: computed tomographic findings and clinical consequences. J Comput Assist Tomogr. 2011;35(1):86–90. 10.1097/RCT.0b013e3181fce2cb. 21245693 10.1097/RCT.0b013e3181fce2cb PMC3675219

